# The Essentials in the Equipment of a Hospital Laboratory*Read before the staff of the Georgia Baptist Hospital, March 12, 1914.

**Published:** 1914-05

**Authors:** Allen H. Bunce


### *THE ESSENTIALS IN THE EQUIPMENT OF A HOSPITAL LABORATORY.

By Allen H. Bunce A. B., M. D.

Although the hospital laboratory was one of the latest additions to the modern hospital, it is destined to become, next to the operating room, the most important branch of the hospital.

*Read before the staff of the Georgia Baptist Hospital, March 12, 1914.

Merely to name the essentials of the laboratory is not such a difficult matter as it is to properly and profitably use it after we have the essentials. No truer statement was ever made than that costly apparatus and marble rooms are not necessary for the prosecution of scientific medicine. A modest laboratory well arranged, conveniently located, and constantly used is of more value than a much more elaborate one not situated or arranged properly. Indeed much of the best work of not a great many years ago was accomplished by men in small villages or in the country where scarcely any equipment was obtainable. Jenner was practicing in a small village when he tested and demonstrated the value of small-pox vaccination. Some of Pasteur's greatest work was accomplished before he ever saw Paris. Marion Sims was a practicioner in the village of Montgomery, Alabama, when he perfected his operation for vesico-vaginal fistula. Our own Crawford Long was a country doctor when he first used ether for general anaesthesia. In fact, many of our greatest discoveries in medicine were made under very trying and difficult circumstances.

However, I do not wish it understood that the modern hospital should not be well equipped in every particular, especially in its laboratory and operating rooms. A careful selection should be made of that equipment which is necessary and the very best obtainable should be purchased, because here, as well as elsewhere, the best is always cheapest.

The first esential is a good microscope with an oil immersion objective and one or two other objectives of lower magnifications. A mechanical stage is almost indispensable. Besides this is a good electric or water power centrifuge, blood counting apparatus, hemoglobinometer, dark field illuminator, etc. should be added. Of course, glass-ware, such as slides, coverglasses, test-tubes, funnels, graduates, beakers, and a buret are to be included. Not the least important is a carefully selected and prepared list of chemicals, stains, and reagents. This, we might say, includes such equipment as is necessary for the routine examinations of urine, blood, sputum, stomach contents, feces, etc. A simple warming stage, which can be made from a piece of copper sheet by any tinner in a few minutes, is necessary for the examination of feces for amoeba coli. We need these for the correct diagnosis of nearly every medical case and a great many surgical conditions. To put a patient on the operating table except in extreme emergency without a complete urinalysis is nothing short of criminal negligence. Also, to neglect to make those examinations which may throw light upon an otherwise uncertain diagnosis in medical cases is no less to be condemned. It is true, for instance, that an examination of the stomach contents gives us no absolutely diagnostic data in a great many gastric disorders, yet, on account of its absolute value in many others, it should not be omitted.

An incubator and some of the simpler culture media should be included for a bacteriological study of those surgical and medical cases where the nature of the offending organism cannot be determined from simple smear preparations. This could also be used where it is advisable to make an autogenous vaccine in the treatment of suitable cases.

However, that branch of laboratory work which is of great importance to the surgeon is tissue diagnosis. On account of the former delay in all tissue examinations necessitated by the routine methods where the specimens were fixed, hardened, imbedded, cut and stained before even a preliminary examination could be made, causing a delay of several days, tissue diagnosis has not been utilized by the surgeon as much as would have otherwise been the case. The reason for this state of affairs is because the surgeon could not see the value of having specimens examined when he could not get any report until his patient was either well or dead and forgotten, of course this lessened the interest of both surgeon and pathologist, also, the diagnosis, coming at such a time, was of little value to the patient.

But we can see a way out of this trouble in the use of the freezing microtome. Although this had been in use for some time previous, it was not until 8 or 9 years ago that it began to be used constantly in any large hospital for the diagnosis of tissues while the operation was still in progress.

The all-important question for the surgeon, in all doubtful cases, is to know whether or not he is dealing with a malignancy. Another important question is: How far has the diseased process extended? Are the glands involved, etc.? With the use of the modern freezing microtome and with the aid of a competent pathologist a diagnosis of nearly all the carcinomatous growths and a great many of the sarcomatous can be made with almost absolute certainty within five or ten minutes after the section of tissue is removed. To accomplish this the histological laboratory must be located adjacent to the operating room so as to economize time. Either the Spencer or Leitz microtome may be used and carbon dioxide is used for freezing. (At the Mayo Clinic the Spencer is used. Personally I use the Leitz and the Leitz is also used by me at one of the private hospitals in town, where it was installed by one of the *surgeons for use in his cases.)

For the preparation of tissue for the freezing microtome, I have used both the rapid fixation method with formalin and also the fresh tissue without any fixation. Of these I prefer the following method of Dr. L. B. Wilson (Journal A. M. A., Dec. 1, 1905) for preparing and staining sections, since it is the most satisfactory of any where quick tissue diagnosis is desired.

"1. Fresh tissue, the cells of which must still be alive in bits of not more than 2x10x10 mm. is frozen in dextrin solution on a good microtome and cut in sections 5 to 15 microns thick.

"2. Remove the sections from the knife with the tip of the finger and allow them to thaw thereon, thus avoiding later air bubbles.

"3. Unroll the sections with a camel's hair brush in 1% NaCl solution.

"4. Stain 10 to 20 seconds in Unna's polychrome methylene blue, Grubler's.

"5. Wash out momentarily in 1% NaCl solution.

"6. Mount in Brun's glucose medium."

Unna's polychrome metheylene blue gives a good differential staining to the various tissue elements, varying from red to purple to dark blue. It is the most satisfactory stain I have found for this purpose.

This equipment for quick tissue diagnosis is just now, I think, one of the most important things to be included in our laboratory equipment.

In addition to these materials, should be included a suitable room for a museum where specimens of interest could be permanently preserved, also if possible, rooms for the photographing of gross specimens and of microscopic sections so that a permanent record may be kept of each specimen.

Finally, of all those things which should be considered in the establishment of a modern hospital, there is none which deserves more careful thought, and further, there is none which will reap more justly earned praise from both physician and patient than a well equipped and scientifically conducted Hospital Laboratory.

214 Grand Opera House Building.

*Dr. E. C. Davis, at the Davis-Fischer Sanatorium.

